# Creative adapting in a fluid environment: an explanatory model of paramedic decision making in the pre-hospital setting

**DOI:** 10.1186/s12873-018-0194-1

**Published:** 2018-11-15

**Authors:** Gudrun Reay, James A. Rankin, Lorraine Smith-MacDonald, Gerald C. Lazarenko

**Affiliations:** 1 Faculty of Nursing, University of Calgary, 2500 University Dr. NW, Calgary, AB T2N 1N4 Canada; 2 Alberta Health Services, Seventh Street Plaza, 14th Floor, North Tower, 10030 – 107 Street NW, Edmonton, Alberta, T5J 3E4 Canada

**Keywords:** Decision-making, Paramedics, Paramedic judgement, Grounded theory, Emergency Medical Services, Pre-hospital care

## Abstract

**Background:**

Paramedics work in a highly complex and unpredictable environment which is characterized by ongoing decision-making. Decisions made by paramedics in the prehospital setting have implications for patient safety, transport, treatment, and health resource utilization. The objective of this study was; a) to understand how paramedics conduct decision-making in the field, and b) to develop a grounded theory of paramedic decision-making in the prehospital setting.

**Method:**

This study was conducted using classical grounded theory. Paramedics (*n* = 13) with five or more years’ experience, who worked in a large urban center in Western Canada were interviewed. Field observations were conducted, each lasting 12 h, with five different ambulance crews. The data were analyzed and coded using the constant comparative method.

**Results:**

The resultant theory, *Creative Adapting in a Fluid Environment,* indicates paramedic decision-making is a fluid iterative process. Unpredictable and dynamic features of the prehospital environment require paramedics to use a flexible and creative approach to decision-making. The model consists of the three categories *constructing a malleable model, revising the model,* and *situation-specific action.* Two additional components, *safety* and *extrication,* are considered at each stage of the call. These two components in conjunction with the three categories influence how decisions are made and enacted.

**Conclusion:**

Paramedic decision-making is highly contextual and requires accurate interpretation and flexible cognitive constructs that are rapidly adaptable. Evaluation of paramedic decision-making needs to account for the complex and dynamic interaction between the environment, patient characteristics, available resources, and provider experience and knowledge.

## Background

In contrast to the hospital Emergency Department (ED) context, paramedics conduct decision-making (DM) in the uniquely complex and unpredictable prehospital environment. Decision-making by paramedics is a multifaceted process, the outcome of which has implications for patient safety and health resource utilization. To date, paramedic DM is poorly understood. Few studies have been conducted on paramedic prehospital DM [1]. A process map constructed by Jensen et al. [2] based on researcher experience and a review of the literature showed that most decisions occur at the scene prior to transport. Jensen et al. [2] findings indicate the need for further exploration of prehospital paramedic DM.

The prehospital setting is the scene where paramedics interact with the patient. Campeau [3], who conducted a grounded theory study of 24 Canadian paramedics, noted that every-day places must be converted into functional workspaces by paramedics on an impromptu basis. Features of the scene thus affect how paramedics make and enact decisions. Furthermore, the scene can provide vital clues as to the mechanism of injury or underlying pathology that, in conjunction with physical patient assessment data, form the basis for DM and treatment. For this to occur clues have to be interpreted correctly, in other words the paramedic’s situation awareness needs to be accurate [4]. For example, insulin syringes may indicate a hypoglycemic reaction and provide a reason for confusion in a patient. However, if environmental clues are interpreted incorrectly resulting in faulty situation awareness then the wrong assumptions may be made [4]. Hodell et al. [5] reported that, for example, an acute stroke can be misconstrued as an overdose if the setting suggests a drug use environment.

On-scene emergency care consists of rapid patient assessment, determining acuity level (triage level), evaluation of treatment options, scene management, and decisions related to patient transport [1, 3, 6]. It has been suggested that paramedics do not use rigid DM tools [1, 7]. Findings from two US studies of paramedic DM when responding to trauma calls indicate that paramedics initially rely on visual cues and what they call “gut feeling” when making on-scene decisions [1]. Jones et al. [1] conducted nine focus groups with EMS providers (*n* = 50) in the Eastern US. Participants discussed “initial instincts” and “gut feeling” when they described scene and patient assessment [1]. The investigators concluded that paramedics used past experience and “Gestalt impression” [8] within the first minute to make decisions that predominantly influenced their choice in destination hospital. These findings were reiterated by Newgard et al. [6] in an ethnographic study (*n* = 35) aimed at understanding cognitive processes of paramedics. They reported that EMS providers formed rapid, heuristic decisions based on information from the dispatcher, visual cues gleaned from the scene and patient, and their experience [6]. In both studies the authors concluded that triage trauma guidelines do not match the order in which paramedics process the flow of information, because guidelines are static and prescriptive and therefore not compatible with a dynamic environment.

Provider judgment is recognized as a critical aspect of paramedic DM. For instance, provider judgement is included as one criterion for field based trauma activation in the US national guidelines for field triage of injured patients [9]. The guidelines contain a four step algorithm organized according to physiologic criteria (e.g. GCS ≤ 13), anatomic criteria (e.g. penetrating injury to torso), mechanism (e.g. ejection, partial or complete, from automobile), and special considerations (e.g. age, provider judgement) [9]. Results from a multi-region retrospective cohort study in the US showed that out of 46,414 EMS calls in which trauma activation was initiated, EMS provider judgement was the most commonly cited criterion used in 25.7% of the calls [9]. The intent of the study was not to determine paramedic accuracy in the use of the trauma guidelines, rather to assess and compare the use of guidelines between different regions. The results do, however, point to the frequent use of paramedic judgement when making decisions at the prehospital scene.

Not surprisingly, compared with inexperienced paramedics, experienced paramedics have been found to rely less on DM tools and more on provider judgement [6, 7]. Experience and knowledge are used to interpret information gathered from the scene and patient [6, 7]. Arbon et al. [7], conducted focus groups and interviews with 14 Australian paramedics. They reported that, although the paramedics had difficulty articulating their DM process at times, they used their experience, insight, and education to formulate decisions; a process collectively described a “gut feeling” [7]. Paradoxically however, Fairbanks and colleagues [10]. reported that the most common reason for adverse events cited by paramedics were errors in judgement.

The previous research that focused on trauma DM indicates that paramedic judgement is an essential component of paramedic DM in general. Paramedic work and DM, however, involves a wide range of calls and is not limited to trauma DM. It is therefore equally important to understand how paramedics formulate and prioritize their decisions for all types of calls as this has implications for patient safety, efficient prehospital care, and professional training. The above review of the literature suggests that paramedic judgement, patient characteristics, and features of the scene are critical factors in paramedic DM.

### Importance

To date, paramedic DM is poorly understood and few studies have been conducted on DM by paramedics in the field [1]. Decision making by paramedics is a complex process, the outcome of which has implications for patient safety, provider safety, and health resource utilization. [1, 2, 6, 7, 11]. Therefore, understanding the cognitive processes of paramedics as they formulate decisions in the field is vital for safe and efficient patient care, reducing error, supporting optimal work processes, and future development of DM support tools.

### Goals of this investigation

The specific research objectives in this qualitative study were to understand the cognitive processes of paramedics as they conduct DM in the prehospital context for all types of calls and to generate an explanatory model of paramedic DM. To meet this objective, we used the grounded theory (GT) research method to analyze data from observations and interviews with paramedics.

## Methods

### Study design and setting

The investigators determined that the GT method, that includes observations and interviews, would be most appropriate in addressing the primary research objective of understanding paramedic DM in the field. GT is primarily an inductive research method that is used to understand what is occurring in a specific social context derived from the perspective of individuals in that milieu; the goal is to generate an organizing concept or theory [12–14]. The researcher does not seek to validate a pre-existing theory; rather the objective is to inductively generate an explanatory ‘grounded theory’ of the topic under investigation. It is important to emphasize that ‘grounded theory’ is also the name given to the qualitative research method.

We designed the study in accordance with GT principles using the constant comparative method for data analysis [14]. Data collection and analysis therefore occurred simultaneously through an iterative process. Initial coding resulted in preliminary codes inductively derived from the data, which were then used to refine questions and elicit points for observation for subsequent rounds of data collection [15]. Emerging clusters of codes, so-called categories, were continually tested against the data for fit during each round of data collection and analysis [15]. Through a process of reduction, and increasingly higher levels of conceptualization, categories were further collapsed. Finally, we used a deductive approach to explore relationships among the remaining categories until an organizing theory of paramedic DM was generated [12].

The study was conducted in a city of over one million people in Western Canada where EMS are part of the publicly funded provincial healthcare authority. Emergency calls for medical assistance are received by a central dispatch centre staffed by non-medical personnel. Dispatchers use the medical priority dispatch system (MPDS), which governs how they answer and ask questions during a 911 call [16]. They follow a strict algorithm and calls are organized according to a card system containing diagnostic criteria. A computerized system notifies paramedics when they are initially dispatched, and information is continuously updated by the dispatcher.

Paramedics use the card systems as a guide, however the treatment that is provided is based on their own assessment, limited use of diagnostic equipment, and history taking. All paramedics are required to use a set of evidence-based Medical Control Protocols from the healthcare authority that have been reviewed by emergency physicians and paediatricians [17]. Depending on the findings, paramedics decide which protocol to follow, or may decide to use several protocols concurrently. If a situation occurs ‘outside’ of their protocols, advice is needed, or certain medications need to be administered, they are required to contact a designated on-call physician for advice. From April 2015 – March 2016, paramedics responded to 111,344 calls and 73,381 patients were transported to a healthcare facility.

### Selection of participants and data collection

We asked EMS managers to forward an email to all paramedics within the study zone (*n* = 495) and to display a recruitment poster at EMS stations. Interested participants (*n* = 25) were asked to contact the principal investigator (GR) directly, who screened them for eligibility. Individuals with five or more years’ experience as a paramedic in non-supervisory positions were eligible to participate (*n* = 13). Data collection occurred from March 2016 to October 2016.

GR conducted all interviews in a private location at the University of Calgary. Interviews lasted 45 to 60 min, were digitally audio recorded, and transcribed by a professional transcriptionist. During the interviews, GR took notes and the transcripts and notes were entered into software known as NVivo11.2.1 [18]. NVivo 11.2.1 software supports qualitative research by facilitating the organization and analysis of unstructured data obtained from interviews.

We prepared a set of guiding questions (see Appendix A), however, the interviews were conducted in a conversational style to encourage a natural flow of information as per the GT method [13]. When an issue surfaced that was not in the question set the interviewer explored and clarified the topic with impromptu questions. The questions were refined after each interview to reflect what participants considered to be vital to their work and DM (see Appendix B). This process allowed us to remain sensitive to emerging concepts [12]. We conducted field observations by so-called ‘ride-alongs’, which entailed following an ambulance crew (GR three, JR one, and LSM one) for an entire 12-h shift focusing on how paramedics worked with the information that was available to them about the patient. In total, five ‘ride-alongs’ were conducted and the team observed 22 calls. Each observer took field notes, which were transcribed by the observer into Word 2011 immediately after the ride-along. It is possible that having a researcher present may have influenced how the call was conducted, however, the research team met following each ride-along for analytical discussions of the observations. The data from the observations were found to be consistent with the data collected during the interviews. Conducting field observations allowed the research team to observe how paramedics elicited the clinical data they needed, how the physical environment influenced decisions, how they worked with treatment protocols, and how they made decisions about destination hospitals.

Ethics approval was obtained from the Conjoint Health Research Ethics Board at the University of Calgary (REB certificate 15–2715). Written informed consent was obtained prior to each interview. Participants were informed that participation was voluntary and they could withdraw at any point before data analysis commenced. Each participant chose an alias that was used in the written documentation. They were advised that no participants could be identified in subsequent publications or presentations. Participants were reimbursed for mileage and received a $10 coffee card.

Paramedics signed written informed consent prior to each ride-along. The major focus was observation of the paramedics; however, for obvious reasons, the investigators came into close contact with patients. Prior to the ride-along we asked paramedics to inform patients, as soon as conditions allowed, that we were researchers observing their work. Patients were advised that no identifying data would be collected and that they could refuse to have the researcher present; none of the patients refused. For patients with altered level of consciousness we obtained approval from next of kin.

### Data analysis

Data collection yielded a total of 400 pages of field notes, interview notes, and interview transcripts, which were stored on a secure university server. Following each interview and observation data were entered in NVivo 11.2.1, coded and analyzed before the next occasion of data collection. This process allowed us to remain sensitive to emerging concepts, to refine interview questions, and conduct progressively more focused observations [12]. The team discussed emerging concepts and hypothetical conceptual relationships throughout the coding process and theoretical memos were written as the coding progressed. Initially line-by-line open coding was used naming and comparing incidents (codes) to each other. [15] Similar incidents were grouped into categories, which were progressively refined and collapsed into more comprehensive categories through a process of reduction [12]. Following eight interviews and four observations the category *adaptation* had emerged as a potentially organizing concept with six possibly related categories of *safety, extrication, scene, resources, critical finding,* and *working with a model.*

Selective coding was used to code the transcripts and field notes for only those categories that related meaningfully to our organizing concept [12]. The six relating categories identified by the team were continually revised. Some categories were discarded and others were expanded. For example, *the scene* was discarded as a separate category and instead integrated into the other categories, whereas *working with a model* was expanded into three categories. At this stage of data collection, the team agreed that theoretical sampling should be used during interviews and observations to fully saturate categories that were identified as theoretically relevant to the organizing concept [14]. Interview questions and observations therefore became more targeted. Data collection ceased when we reached data saturation; the point when no new data emerged about the relevant categories [12]. Throughout the data analysis we created visual representations of paramedic DM based on the data. These representations, which illustrated our emerging DM model, were continually evaluated by the team and refined as data analysis progressed. Finally, through a series of discussions among team members exploring hypothetical relationships among categories and the organizing concept, an explanatory model of paramedic DM and was generated.

## Results

### Characteristics of study subjects

We interviewed 13 paramedics in non-supervisory positions (five women and eight males) whose work experience ranged from 5 to 29 years (M = 13.3 years). Sixty hours of field observations were conducted for a total of 22 EMS responses.

### Main results

**Fig. 1:**
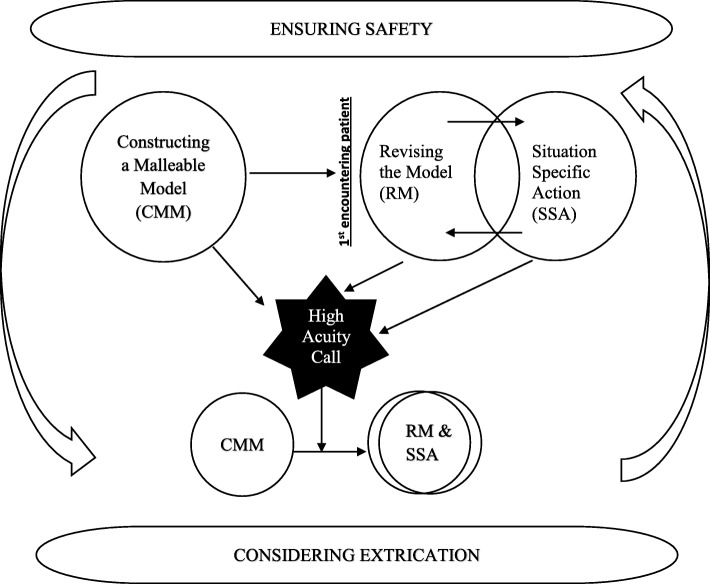
EMS Decision-Making Conceptual Model

Our data revealed that the organizing concept for paramedic DM in the prehospital setting is a process of *creative adapting in a fluid environment.* As can be seen in Fig. 1 (EMS decision-making conceptual model*)* Creative Adapting consists of two overarching components, *ensuring safety* and *considering extrication.* Decision-making is not a static linear process, it is a fluid situation dependent process in which safety and extrication are considered during all the stages of the call (*constructing a malleable model, revising the model, and situation specific action)*. Paramedics construct a malleable model of the call based on information supplied by the dispatcher and information gathered from the scene even prior to engaging the patient. On encountering the patient, they revise their model and initiate situation specific actions. As may be seen in Fig. 1 there is overlap between revising the model and situation specific actions. Patient and scene conditions necessitate that paramedics enact decisions while continuously modifying the mental construct with which they work. A high acuity call results in an extremely condensed version of the model.

It is emphasized that no model or theory can fully capture the complexities inherent in providing prehospital care. Paramedics work in a dynamic environment, each call includes a unique combination of patient and location features, which requires on the spot creative adaptation to the specific context. However, to provide a foundation for understanding and discussing DM by paramedics we provide a model derived from observations and participant accounts. In the subsequent sections, we describe the categories. Representative quotes from the participants are provided in Table 1.

**Table 1:** Quotes Describing our Model of Paramedic Decision-Making

Ensuring Safety		
*I recently went on a call where it was a house that my partner and I had been to before that was a very sketchy drug house that we’d had a bad experience at before. And as soon as we rolled up we recognized the house and went no, no; we’re not going in. But it was you know like a gentleman in his forties calling for chest pain, so it could have easily been a STEMI (ST-elevation myocardial infarction), so its hard sometimes to make that decision of you know, should we go in because if this guy is having an actual heart attack or something (Participant #5)*		
Constructing a Malleable Model	Revising the model	Situation Specific Action
*Prepatory cognition* *Sometimes you can tell what this is going to be; you know just based on the location, based on a couple of the notes, you know how this is going to be nothing. Or you read the notes and you go this is going to be a STEMI (ST-elevation myocardial infarction). I can tell this is going to be a STEMI. (Participant #4)* *Looking for clues* *You look for things like alcohol. You pay attention to if there are any smells. Is there a pleasant odour or a fecal smell. Are there any drugs? You essentially use all your senses. All this happens in the first few milliseconds. It is not a step by step process. There are things you recognize and pay attention to. You notice what you hear, see, and smell. You have already assessed the environment as you meet the patient and you have already started to evaluate what might be going on. (Participant #13)*	*Initial impression* *I look at colour, how are they breathing, what is their work of breathing, is their breathing distressed, are they conscious, are they tracking with their eyes, their facial expression, do they look distressed? (Participant #7)* *Initial determination* *I consider whether the diagnosis is supported by the signs and symptoms, the environment and the history and then I go with the most likely. If I don’t have enough information I go with the most responsible diagnosis. (Participant #10)* *You look for clues about how they’re living. I can’t walk. You’re on the second floor of your house in the bedroom. How have you gotten around all day? Well, I walked up here. (Participant #4)*	*Reasoning about treatment decisions* *If I think they already have drugs onboard and they are already drowsy and say they have 10/10 pain I won’t give drugs, but I make sure I can justify it if I get called in. Things like they were already drowsy, there was pot in the home, I was concerned they had something else onboard (Participant #8).* *Working with protocols* *Okay, in this given situation, this is what you’re going to have to do. And the way they’re written, they’re kind of written in a linear form and box A followed by box B and box C. That doesn’t always work depending on the patient. Sometimes you’re going to have to mix and match around a little bit. (Participant #2).* *Seeking medical advice.* *I have always stayed within my scope of practice and where it is appropriate call online medical back up But we know that could take 10–15 min. And when someone is hypoxic and you need orders you need them now. You don’t have 20 min to tell the Dr. the story when you wake him up in the middle of the night. This is what I have, this it what I need, can I go ahead. The Drs. also don’t like being asked if they don’t know who you are. (Participant #7)* *Initiating treatment* *We had an adult woman with a dislocated shoulder up in the far corner of a kids jungle gym, 30 ft in the air and there was no moving her as we had to crawl and weave through obstacles, walls etc to get her down. We had to use the fire department to help us, we got orders from our physician to sedate her with fentanyl and ketamine and attempt to reduce her shoulder there on the spot. We were 30 ft in the air a long ways from our ambulance so we had ALL of our equipment there, all of our airway management stuff, medications, monitor etc just in case things went poorly. (Participant #4)*
Considering Extrication		
*Here’s another gray zone. I had a gentleman that was---if I recall he was seven hundred plus pounds. He was in a-fib, RVR, at a rate of like a hundred and thirty and we had to get him outside and take him to the hospital. We can’t carry that. Should he be carried? Of course he should. He’s seven hundred pounds with a heart rate of a hundred and forty plus in a-fib. Walking him is not a good idea, but how do you get him out of the house? (Participant #4)*		

### Ensuring safety

Two aspects of safety, personal safety and patient safety, permeate the work of paramedics. When paramedics arrive at a location they assess their personal safety; they delay entering the scene if it is deemed unsafe. Examples of unsafe scenes include, a known “drug house”, aggressive dogs, intoxicated bystanders, environmental hazards, dangerous traffic conditions, the presence of weapons, and confrontational patients. Upon entering a scene paramedics remain vigilant and continuously assess their surroundings to obtain a gestalt of hazards; they ensure that they are aware of all exit routes. At this stage ensuring safety is about personal safety, however, the indicators they consider become part of the malleable mental model they have begun to construct of the call.

Paramedics are cognizant of personal and patient safety when they assess patients. An agitated or aggressive patient jeopardizes their safety and therefore results in an abbreviated assessment and revision of treatment options. Features of the scene, such as aggressive family members or toxic substances, may jeopardize patient safety and therefore lead to immediate removal of the patient prior to initiating treatment. Paramedics delay moving the patient if this might result in injury to themselves or if essential treatments need to be initiated immediately to ensure patient safety. In short, due to safety concerns paramedics revise their model of how the call will unfold and initiate situation specific actions.

### Considering extrication

Moving the patient to the ambulance is referred to as extrication by paramedics. On approaching a scene paramedics consider access and exit points, automatically noting narrow hallways, doors, and stairs that could make transfer difficult. During the assessment, they revise their mental model and consider patient acuity, body habitus, ease of transfer, and staff resources. These factors are incorporated into the decision about extrication. Paramedics adapt their extrication decisions in relation to the scene in conjunction with the initiation of treatment and transport requirements. An unstable patient who, ideally would be extricated without delay, may have a prolonged scene time due to the need for immediate treatment, insufficient numbers of staff for extrication, or complicating features of the scene. Paramedics consider initiating treatment, extrication, and maintaining safety simultaneously and make situation specific decisions in a dynamic environment.

### Constructing a malleable model

Our data show that paramedics work with a flexible mental construct of how the call will unfold. Prior to meeting the patient they use *preparatory cognition*. Information from the dispatcher is considered in conjunction with their medical knowledge combined with their on-the-job acquired knowledge and experience of different scenes. At this point the model they are constructing is tentative and subject to continuous modification.

Paramedics add to the model as they approach and enter the scene by *looking for clues* in the environment. For instance, in a private residence they scan the dwelling for the general state of the home, empty medication bottles, or drug paraphernalia and make inferences about the reason for the 911 call. The participants described this process as being automatic. The paramedics incorporate the clues they find into the patient assessment; their patient assessment is in fact initiated prior to meeting the patient. In this way, the scene and the clues that are generated influence the shape of the paramedics’ working mental model.

### Revising the model

When paramedics meet the patient, they supplement their mental model with specific patient assessment data and make necessary revisions. Findings from our study indicate that the patients were frequently significantly different from what the paramedics had anticipated based on the information received from dispatch. Participants recounted how they make a rapid decision based on their *initial impression*. The patient’s level of consciousness, work of breathing, skin color and condition, and signs of obvious distress were the major parameters that provide vital information about patient status. The *initial impression* concerning the major parameters results in a preliminary decision to establish the acuity of the call.

If the patient does not need immediate life or limb saving interventions, paramedics then make an *initial determination* by conducting a situation-dependent assessment. During the *initial determination,* they consider the underlying pathology or injury, the treatment required, and the need for transport. Through a process of *combining variables, matching variables*, and *questioning inconsistent variables* they mentally reason about the patient’s signs and symptoms and the events leading to the 911 call. Paramedics were found to:

- *Combine variables* by considering medical history, events leading to the 911 call, vital signs, ECG tracings, level of pain, physical findings, clues from the scene, and supplemental information provided by other informants, such as firefighters or bystanders. The actual physical hands-on assessment occurs as they continue to gather information from the patient.
  During the assessment, they reason with themselves about how the variables fit together. They continually adjust the mental model they are constructing by adding new information and revising the model. Variables are combined and evaluated to see if they form a coherent whole. Paramedics seek to understand the underlying pathology by combining their experience, knowledge, and the data gathered at the scene.
- *Match variables* by looking for consistency among the various data sources from the patient, environmental clues and other collateral information.
  For example, with a confused patient, paramedics consider the events leading to the call, medical history, the scene, and information from bystanders to determine whether the data matches the patient’s presentation.
- *Question inconsistent variables* when there is no a coherent whole or match. For example, a patient may appear well and have abnormal vital signs. They then mentally reason why this may be the case.

Inconsistent variables can be vague but sufficient to trigger suspicion that their initial determination of the underlying pathology is incorrect. Paramedics refer to this phenomenon as ‘medics nose’ or a ‘gut feeling’. They have identified a variable that is not part of the coherent whole and is inconsistent with what they would expect based on their knowledge and experience.

### Situation specific action

Paramedics have now adapted the previously constructed mental model to scene and patient specific characteristics. For the purposes of clarity, we have described DM in a linear fashion, however, some treatment decisions are made and initiated during the assessment, hence the overlap in our model.

*Reasoning about treatment decisions* is a process in which paramedics use collateral information, assessment findings, experience, and knowledge to theorize about the underlying diagnosis. Interventions are selected based on suspected diagnosis and symptomology. In cases where the underlying pathology is unclear, paramedics may elect to treat symptomatically. Paramedics have a strong sense of being solely responsible for treatment decisions. This is a function of their work environment in which one person is designated “the driver” and the other is “the attending”; the latter oversees patient care and signs the patient care record. Paramedics talked about good working relationships with their partners and working collaboratively, however, they were acutely aware of the need to be able to justify their decisions if adverse events occurred.

Paramedics are required to follow *protocols*. Common protocols are internalized, it is therefore not obvious when observing their work that they are in a protocol driven environment. Protocols are often applied retroactively, sometimes creatively, when they document their findings. We are not implying that paramedics ignore protocols, only that they place significant emphasis on their own knowledge base and the need to adapt treatments to each specific context. There is evident tension when paramedics discuss protocols. On one hand, they consider protocols a good resource; on the other hand, some participants considered them too rigid. If paramedics elect to consciously deviate from protocols, it is founded on the belief that it is in the patient’s best interest.

Paramedics have the option of *seeking medical advice*. This occurs primarily under three conditions; a) medications that require physician approval, b) a patient that requires hospital care who refuses to be transported, and c) the need for treatment advice. The latter is viewed by paramedics as a safeguard against potential ramifications in the event of a “wrong” decision.

In our DM model (Fig. 1), being unsure of the underlying pathology and determining how to proceed with treatment represents a scenario in which their mental model does not fit with the patient presentation. The immediacy of prehospital work and the need for rapid decisions sometimes situate paramedics in a complex DM space.

*Initiating treatment* can occur at the scene or in the ambulance. As with patient extrication, the decision about when and where to initiate treatment is a function of the patient’s condition, scene characteristics, and staff resources available. Paramedics are often required to use creativity in conjunction with experience and medical knowledge to adapt treatment decisions to specific contexts.

### High acuity calls

A high acuity call is the result of a critical finding, defined by paramedics as a condition that requires immediate intervention or immediate change of treatment, for example a cardiac arrest or major trauma. The critical finding represents a cut-off point that interrupts the ‘routine’ aspects of paramedic work. A ‘routine’ call can change into a high acuity call during any of the three stages in our model. As indicated by the participant quotes in Table 2, paramedics rapidly formulate a plan while simultaneously initiating it during a high acuity call.

**Table 2:** High Acuity Calls Quotes

High Acuity Quotes	
*At the house someone came out and said there were three people inside. It instantly throws your whole plan out the window. Now you are going to have to triage as you find the people. Who are you going to treat first and how many more resources are you going to need. The first pt. was apneic with a pulse. I told my partner (EMT) to give him Narcan and protect the airway. Then I went looking for the others. I found two other patients with agonal resps at 4–6/min. Again with the minimal amount of hands and the fire department not being familiar with where we store our meds I made the decision to have them protect their airways while I went out to the truck to get our equipment and Narcan. Meanwhile I had already called for back-up and the fire captain was trying to organize back-up too. (Participant #9)*	
*There is nothing that rips us more than having someone at the hospital not appreciate the busy environment we were in. Having someone hone in on why did you not do this or that. I am sorry we were busy intubating (becomes very animated), doing CPR, giving drugs. You are lucky you got a patch. (Participant #7)*	
*The call came in as someone unconscious in a vehicle. There was an off duty Dr there and a male who had been with her in the car. He thought she had used Fentanyl and heroin. You could tell by looking at her, her breathing was like 4/min. So we got a stretcher, dragged her out, ventilated her, there was another ambulance nearby, they drove. We gave her Narcan. I don’t think I even got an IV. We were at the hospital in like a minute. I patched. Our scene time was like 4 min.* *We could maybe have taken the time at the scene, but it was a respiratory problem as opposed to a cardiac problem. She needed definitive care at the hospital, which was like a 100 ft away. It made no sense to stay when help was so close by. We did minimal amount to keep her alive. (Participant #13)*	

Furthermore, as may be seen from our decision-making model in Fig. 1, this is represented by the near complete overlap of the categories *revising the model* and *situation-specific action*. Paramedics determine the need for and call for additional human resources as they consider extrication in relation to scene and patient conditions. At the same time, they consider events leading up to the 911 call and possible causes, and considering this they implement a treatment plan while continuously revising it.

During high acuity calls knowing your partner is vital to paramedics as they anticipate each other’s actions. The attending assumes the leadership role, which includes directing and assisting with treatments, and delegating tasks to co-workers and firefighters. Expedient extrication and transport is not always possible due to complicating scene or patient factors. For instance, a patient may require life or limb saving treatment, or sedation prior to being moved.

Paramedics expressed frustration with the limited understanding by hospital staff of how the dynamic interplay of variables in the field and the seriousness of the patient’s condition can affect the length of time on scene (Table 2).

In summary, paramedic DM is a highly contextual process which requires them to creatively adapt their decisions to the unique patient and scene features of each call. Safety and extrication considerations influence decisions throughout the call. Paramedics construct a malleable model of how the call might unfold, revise the model when they meet the patient, and institute situation specific actions based on their patient assessment, clinical experience and knowledge, scene characteristics, and available resources. DM is not a linear activity in which protocols are applied in a step-wise fashion, rather it is a complex fluid process which occurs in a dynamic and unpredictable environment.

## Discussion

We used GT to develop a practice-based explanatory model of paramedic DM that provides a conceptual framework for understanding how paramedics make decisions. The results expand on previous studies in which paramedic DM and EMS provider judgment have been investigated. Our model makes explicit that paramedic DM is a complex and dynamic process that requires them to make decisions on an ongoing and iterative basis. Furthermore, the results demonstrate that DM is not linear, rather there are multiple levels (micro, meso, and macro) and factors that paramedics consider throughout the call which constantly impact their DM process.

Similar to previous research we found that paramedics use information received from the dispatcher in conjunction with their knowledge of the location as they begin to construct a mental model of the call and anticipate potential actions [1, 6, 7]. Others have noted that paramedics recognize that their assumptions are provisional and may be subject to change once more complete information is available at the scene [7, 19]. Differently stated, the paramedics are constructing a malleable model that is continuously revised as new information emerges.

The scene is central to the work of paramedics; as a source of information about the patient and as a work area that impacts how and when medical procedures are performed [3, 7]. It is this dual function of the scene that makes paramedic work unique. On the one hand and, in contrast to hospital staff, paramedics have access to rich environmental clues that are incorporated into their assessment and subsequent DM. On the other hand, conditions at the scene may limit their ability to intervene in an expedient or optimal manner. Thus, the scene and the impact it has on DM, may become a source of dissonance between ED staff and paramedics. Paramedics in our study often expressed feeling misunderstood or judged by other healthcare providers from comments made about their field decisions. Disparate professional lenses and knowledge may thus have implications for how DM is conducted in the field. In fact, Hodell et al. [5] found that some paramedics expressed hesitancy to activate stroke protocols if they had received recurrent negative feedback from hospital staff.

Similar to past research we found that paramedics indicate that they know within the first few minutes if a patient is critical based on their initial impression, environmental clues, and experience [1, 6, 7]. This is a form of the “rule out the worst-case scenario” DM process commonly used by clinicians [20]. However, rapid heuristic DM may also lead to errors in judgement [21], specifically if the mental construct paramedics are working with is not sufficiently flexible. Lowe et al. [22] reviewed the literature on situation awareness and how it affects a clinician’s ability to perform in the ED. There are obvious and significant differences between the prehospital context and the ED, however similarities exist in that they are both multi-stimuli environments where rapid DM is often crucial. Lowe et al. [22] concluded that situation awareness is essential for patient safety. Endersley [4] proposed a definition of situation awareness consisting of the following three domains.

1. Perception - an individual’s ability to discern relevant features in the environment from displays and sensory input.
2. Comprehension - the ability to integrate and understand what the information means in terms of desired outcome of the situation.
3. Projection – the ability to combine data and anticipate the outcome of actions.

Even expert decision makers will make mistakes if their situation awareness is inaccurate or incomplete [4]. For instance, preconceptions may act as a filter that results in a faulty interpretation of the situation. Information overload may exceed an individual’s attention capacity, or clues perceived to be highly salient may capture the decision maker’s attention to the detriment of other pertinent clues [4].

Klein [23, 24] who developed the naturalistic decision-making model (NDM) showed that decision makers in high acuity dynamic contexts construct a story of plausible events leading up to the incident, particularly if their initial impression does not match their expectations. Similarly, paramedics in our study incorporated clues from the scene in their assessment and questioned the patient’s story if the clues and the story did not form a coherent whole, or was not congruent with their expectations. Emergency triage Registered Nurses (RNs) have been shown to use a similar approach of matching the patient’s story against objective assessment findings, the patient’s medical history, and sensory clues [25]. An integral part of the professional roles of paramedics and RNs is to question inconsistent variables.

Given that the highest decision density during an EMS call occurs at the scene [1] and that the prehospital setting is a multi-stimuli, fluid environment, it is essential that paramedics are aware of the consequences of rigid preconceptions. In addition, it is important that they are cognizant that attending to the ‘wrong’ clues may negatively affect DM, and that information overload can lead to errors. Our findings make explicit that pre-hospital DM is an iterative process that requires practitioners who are flexible in their thinking and who actively question environmental clues that are inconsistent with their assessment findings. While the primary focus of this study was not on how paramedics *use* protocols, our results nonetheless support that paramedics use protocols when making decisions as indicated by them in the interviews. However, during the observations they seldom referred overtly to the protocols because, as they explained, they knew most protocols. Consistent with previous research, our study shows that protocols were used in conjunction with paramedic judgment, experience, knowledge, and what they perceived as intuition [6, 26, 27].

While there was some evidence of hypo-deductive reasoning when paramedics reasoned about treatment decisions, similar to Newgard et al. [26] and Jones [1] we found that paramedic DM does not occur in a stepwise fashion, consequently protocols do not necessarily match how paramedics think. Our participants considered protocols to be objectively useful but felt that they sometimes lacked the flexibility needed in certain practice situations. Paramedic ability to accurately assess medical necessity for transport and injury severity [27] varies considerably across studies, and data does not support the practice of paramedics’ solely using their own judgement and DM skills. The tension between static protocols and the fluid environment will likely continue to pose difficulties for paramedics and policy-makers. We argue that it is healthier and more efficient to explicitly recognize the tension as this may lead to fruitful discussion in the future about increased training and recognition of how paramedic judgment can safely inform the flexible use of protocols.

Our model and conceptualization of paramedic DM as a process of *creative adapting in a fluid environment* brings to the forefront the influence of safety and considerations of extrication throughout the call. Few authors have discussed safety and extrication in relation to prehospital paramedic DM. Campeau [3, 28] and Arbon et al. [7] examined the importance of the scene to paramedic work, however, did not extend the discussion to how safety concerns and extrication considerations influence DM.

Our findings suggest that paramedics conduct a ‘global’ assessment to obtain a ‘gestalt’ of the call. Thus, safety and extrication are not separate entities distinct from patient assessment and treatment, they are features that are considered and incorporated into DM at every stage of the call. Paramedics, therefore, creatively adapt to the unique features of each call. Creatively adapting requires knowledge, experience, flexible cognitive patterns, and accurate situation awareness, which includes perception, understanding, and projection. Understanding the impact of safety issues and extrication considerations on DM provides a more comprehensive lens for untangling how paramedic DM is carried out in the field.

As the focus of this study was to understand how paramedics make decisions in the field from their own perspective, we did not focus on system (macro level) factors which impact their DM. However, paramedics indicated that a primary system factor was their sense of being “excluded” from the larger healthcare system. For example, they do not have access to the electronic patient hospital records and they seldom receive feedback on the effectiveness of their treatment decisions. Similar to the findings of O’Hara et al. [19] participants in our study expressed feelings that communication with other healthcare professionals was sometimes difficult. This included feeling that their professional knowledge was undervalued by triage nurses, and that they were chronically judged and misunderstood by ED staff [29].

Participants expressed a wish to receive constructive ‘follow-up’ from hospital staff and physicians to know whether their treatments were effective. Paramedics expressed this desire particularly when they had to question inconsistent variables or provide only symptomatic treatments. Our participants viewed receiving ‘follow-up’ as an effective way to develop their experiential knowledge and improve their DM process. Feedback was particularly important to paramedics in the setting of high acuity calls (see quotes) when they were required to make quick decisions.

There are several limitations to consider in this study. First, the findings are from a select geographical location in Canada within a publicly funded healthcare system, therefore the applicability of our findings to other EMS providers and healthcare systems must be viewed with caution. Second, our sample was limited to paramedics with five or more years’ experience, therefore we cannot comment on how DM process may be different for Emergency Medicine Technicians and newly graduated paramedics. Third, findings from qualitative research are not generalizable in the same way that quantitative researchers seek to do; however, GT research has the potential to be *conceptually* generalized, meaning that the concept *creative adapting* has the potential be used to understand DM in other high acuity contexts.

## Conclusion

In summary, paramedic DM is highly contextual and therefore needs to be understood as a function of the interaction among wider system influences, environment factors, patient characteristics, available resources, and provider experience and knowledge. Viewing paramedic DM as a linear process in which protocols are applied in a step-wise fashion limits the understanding of the complexity inherent in their DM and the creativity that is essential to making decisions in the dynamic and unpredictable prehospital environment. Recognizing the complexity and fluidity inherent in the work of paramedics has implications for how paramedic treatment decisions are understood and evaluated. Paramedic and EMS training needs to support the development of safe, flexible cognitive constructs. Evaluation of treatment decisions needs to include recognition of how context specific factors affect and are incorporated into decisions. Furthermore, paramedics reported feeling that their professional judgement was undervalued, paramedic DM may be enhanced by establishing mutually respective relationship between ED staff and paramedics with structured processes for constructive feedback on treatment decisions.

## Abbreviations

DM: Decision-Making
ED: Emergency Department
EMS: Emergency Medical Services
GT: Grounded Theory (A research method used to determine underlying processes related to the phenomena of interest)
RN: Registered Nurse
